# Camera Calibration Robust to Defocus Using Phase-Shifting Patterns

**DOI:** 10.3390/s17102361

**Published:** 2017-10-16

**Authors:** Bolin Cai, Yuwei Wang, Keyi Wang, Mengchao Ma, Xiangcheng Chen

**Affiliations:** 1 Department of Precision Machinery and Precision Instrumentation, University of Science and Technology of China, Hefei 230026, China; cbl37@mail.ustc.edu.cn (B.C.); jiyuer@mail.ustc.edu.cn (Y.W.); 2 Department of Instrument Science and Opto-Electronics Engineering, Hefei University of Technology, Hefei 230088, China; mmchao@hfut.edu.cn; 3 School of Automation, Wuhan University of Technology, Wuhan 430070, China

**Keywords:** defocus, camera calibration, phase-shifting, feature points, concentric circle

## Abstract

Camera parameters can’t be estimated accurately using traditional calibration methods if the camera is substantially defocused. To tackle this problem, an improved approach based on three phase-shifting circular grating (PCG) arrays is proposed in this paper. Rather than encoding the feature points into the intensity, the proposed method encodes the feature points into the phase distribution, which can be recovered precisely using phase-shifting methods. The PCG centers are extracted as feature points, which can be located accurately even if the images are severely blurred. Unlike the previous method which just uses a single circle, the proposed method uses a concentric circle to estimate the PCG center, such that the center can be located precisely. This paper also presents a sorting algorithm for the detected feature points automatically. Experiments with both synthetic and real images were carried out to validate the performance of the method. And the results show that the superiority of PCG arrays compared with the concentric circle array even under severe defocus.

## 1. Introduction

Camera calibration is always the first and irreplaceable process in vision systems such as three-dimensional (3D) measurement, microscopy and robot navigation [1,2,3]. Since the calibration accuracy directly influences the performance of the systems, numerous calibration methods have been put forward and various targets have been designed during recent decades. These calibration methods can be roughly divided into two categories: object-based calibration and self-calibration. Also there are three types of objects according to their dimensionalities, namely 3D target [4], 2D target [5,6], and 1D target [7]. Self-calibration does not need any designed targets, yet its accuracy is limited in presence of noise [8].

To our knowledge, 2D targets have been employed widely due to their ease to manufacture and flexibility to use. There are two common patterns of 2D target: grids [9,10] and circles [11,12,13,14,15,16]. By comparison, the circle pattern has been becoming the research hotspot due to its rich geometric properties and high recognition. The earliest idea directly used the center of projection ellipse as the center of a spatial circle. However, as we know, the idea is improper under general perspective [15]. To estimate the real projected centers of circle patterns precisely, Kim et al. [17] proposed a simple method based on concentric circles, with known radii information. After that, many researchers have concentrated on finding various geometric or algebraic constraints of concentric circles to estimate the camera parameters. Zhang et al. [18] presented a solution to efficiently recover the projections of the circle center from the concentric images. The problem is formulated into a first order polynomial eigenvalue problem by considering the pole-polar relationship. Subsequently, Chen et al. [19] suggested a method of calibration based on a planar pattern containing concentric circle array. The imaged centers of concentric circles are located using the principles of the cross-ratio and the pole-polar. To generally improve the computational efficiency, Yang et al. [20] introduced a method only using four intersections and two cross-ratio equations to solve the imaged centers with the concentric circle array. 

These calibration methods are primarily developed for short-range vision systems [21]. The accurate calibration requires the well-focused pattern images. However, if the targets are applied in the long-range vision systems, the camera is usual out-of-focus. Thus, the calibration result is not reliable. In this scene, if the systems require highly accurate calibration results, the targets should be large enough to ensure that the captured images are sharp enough [22]. Evidently, it is a great challenge for long-range systems since fabricate large targets are becoming difficult in terms of accuracy, feasibility, and cost.

To tackle the problem, researchers have designed the targets whose feature points are encoded into the phase domain. With these targets, accurate calibration results can be achieved even if the images are blurred. Schmalz et al. [23] performed camera calibrations with horizontal and vertical phase-shift sequences whose phase distribution was robust against defocusing. Huang et al. [24] employed eight-frame phase-shifted fringe patterns as active targets and further improved calibration accuracy. Bell et al. [21] utilized a set of horizontal and vertical phase-shift fringe patterns to calibrate an out-of-focus camera and An et al. [22] used Bell’s method to calibrate a large-range structured light system. Definitely, the above methods often required that multiple images should be captured at each camera pose and demanded more human interaction, which are laborious and inefficient. In our previous works [25,26], we proposed a method to calibrate the camera with defocused images, but directly used the center of projection ellipse as the real center of a phase-shift circular grating, which can be improved. Since the projective of a circle is not invariant, the circle center which is recovered directly from the center of the projection ellipse is not the real projection [15]. 

In this paper, in consideration of the accuracy of real center location and the application of the target in long-vision system, we propose an efficient approach utilizing the phase-shifting circular grating arrays to calibrate the camera even with the defocused images. We formulate the feature extraction as a concentric circle issue to estimate real imaged centers of PCGs rather than directly using the centers of projection ellipse as the real centers. Thus, the imaged center can be located accurately. Instead of a set of phase-shift patterns, we just need three frames at each pose, which can reduce the workload and improve the efficiency. The wrapped phases are calculated by the three-step phase-shift algorithm [27]. Zero-phase points are roughly detected by Canny algorithm and optimized for sub-pixel precision. We evaluate the performance of the proposed method on synthetic and real data. Moreover, in the contrast experiment with concentric circle array pattern, the proposed method shows its superiority of high accuracy and insensitivity to image defocusing.

Section 2 explains the related works of the proposed camera calibration method, including the camera model, the circle projection model, the pole-polar relationship and the real imaged center estimation. In Section 3, the proposed method is presented. Experimental results on synthetic and real data groups are showed in Section 4. Lastly, Section 5 gives a brief conclusion.

## 2. Related Works

### 2.1. Camera Model

The camera model is a set of mathematical equations that describe the relationship between a 3D world point and its projection onto the camera images plane. For a 3D point $P=\left(X_{W}\;Y_{W}\;Z_{W}\right)$, its corresponding image point is p=(u  v). $\tilde{P}$ and $\tilde{p}$ denote their homogenous coordinates. The imaging process can be simplified as:

(1) $$s\tilde{p}=K[R\;t]\tilde{P}$$

here *R* and *t*, called the extrinsic parameters, represent the rotation and translation matrix from the world coordinate system to the camera coordinate system, respectively; *s* is a scale factor; and *k* is the intrinsic matrix that can be denoted as:

(2) $$K=\left[\begin{array}{ccc} f_{u} & \beta & u_{0}\\ 0 & f_{v} & v_{0}\\ 0 & 0 & 1 \end{array}\right]$$

where *f_u_* and *f_v_* are the focal lengths of the camera along *u* and *v* directions, respectively; *β* is the aspect factor, and (*u*_0_, *v*_0_) is the principal point. If the camera lens is nonlinear, the distortion coefficients can be modeled as $D={\left[\begin{array}{ccccc} K_{1} & K_{2} & p_{1} & p_{2} & K_{3} \end{array}\right]}^{T}$, where *k*_1_, *k*_2_ and *k*_3_ are the radial distortion coefficients, *p*_1_ and *p*_2_ represent the tangential distortion coefficients. For simplicity, the radial distortion coefficients *k*_1_ and *k*_2_ are considered, since the distortion function is mainly dominated by the radial components [5,6].

### 2.2. Circle Projection Model

The common expression of a spatial circle is ${\left(x-x_{0}\right)}^{2}+{\left(y-y_{0}\right)}^{2}=r^{2}$, which can be expressed in matrix from as:

(3) $$\left[\begin{array}{ccc} x & y & 1 \end{array}\right]C{\left[\begin{array}{ccc} x & y & 1 \end{array}\right]}^{T}=0\;with\;C=\left[\begin{array}{ccc} 1 & 0 & -x_{0}\\ 0 & 1 & -y_{0}\\ -x_{0} & -y_{0} & x_{0}^{2}+y_{0}^{2}-r^{2} \end{array}\right]$$

where (*x*, *y*) is the point on the circle; (*x*_0_, *y*_0_) is the circle center; *r* is the radii. Similarly, a 2D ellipse curves $ax^{2}+by^{2}+cxy+dx+ey+f=0$ can be presented in equivalent matrix form as:

(4) $$\left[\begin{array}{ccc} x & y & 1 \end{array}\right]E{\left[\begin{array}{ccc} x & y & 1 \end{array}\right]}^{T}=0\;with\;E=\left[\begin{array}{ccc} a & c/2 & d/2\\ c/2 & b & e/2\\ d/2 & e/2 & f \end{array}\right]$$

Obviously, the spatial circle *C* is in world space, but its circumference distributes on *x*-*y* plane (*z* = 0). The ellipse curves as the projections of the spatial circle are on the image plane. So the matrix from of them can be written as:

(5) $${\tilde{P}}^{T}C\tilde{P}=0$$

(6) $${\tilde{p}}^{T}E\tilde{p}=0$$

Combining Equations (1), (5) and (6), the transformation relationship between the spatial circle and its projection ellipse curves can be obtained:

(7) $$sE={\left(K\left[\begin{array}{cc} R & t \end{array}\right]\right)}^{-T}C{\left(K\left[\begin{array}{cc} R & t \end{array}\right]\right)}^{-1}$$

### 2.3. Pole-Polar Relationship

For a spatial circle *C*, in the same plane, there exists a relationship between a point *p* and a line *l*:*l* = *Cp*. The point *p* is the pole of *l* with respect to *C*, and the line *l* is the polar of *p*. Furthermore, if *p* is the projection of the circle center, and *l* is the intersection line (vanishing line) of the supporting plane with the plane at infinity. In the image plane, *E* is the projection conic of a spatial circle. Then the formula can be obtained as followed [28]

(8) l=λEp

where λ is a constant factor.

### 2.4. Circle Center Estimation

As we know, the circle center cannot be recovered directly from the image for its projective is not invariant. Therefore, the way treating the centers of projected ellipses as the real imaged centers is unreliable. In the literature, the real imaged center could be computed from geometric, algebraic as well as the pole-polar relationship constraints on the projection of concentric circles [18,19,20,29]. Here, we estimate the real imaged center from three PCG images based on the theory mentioned above.

Assuming that $C_{1}$ and $C_{2}$ are the two spatial concentric circles, their projections conic can be $E_{1}$ and $E_{2}$. From Equation (7), we know the transformation relationship between the spatial circles and its projection ellipse curves. So we can obtain the equations as follows

(9) $$s_{1}E_{1}=Q^{-T}C_{1}Q^{-1}$$

(10) $$s_{2}E_{2}=Q^{-T}C_{2}Q^{-1}$$

where $Q=K\left[\begin{array}{cc} R & t \end{array}\right]$; $s_{1}$ and $s_{2}$ are the non-zero scale factors. Subtracting the equations in (9) and (10), we get

(11) $$s_{1}E_{1}-s_{2}E_{2}=Q^{-T}C_{1}Q^{-1}-Q^{-T}C_{2}Q^{-1}=\;Q^{-T}\left[\begin{array}{ccc} 0 & 0 & 0\\ 0 & 0 & 0\\ 0 & 0 & r_{2}^{2}-r_{1}^{2} \end{array}\right]Q^{-1}$$

The radius of two concentric circles is exactly different, so $r_{2}^{2}-r_{1}^{2}$ is a non-zero. The property of similarity transformation notified that the matrix in Equation (11) has a pair of identical eigenvalues, which are different from the third one.

Apparently, the conclusion provides a clue to improve the computational efficiency of solving the circle center. For the concentric circles, assuming its imaged center o and the vanishing line l, From Equation (8), we have

(12) $$l=\lambda_{1}E_{1}o$$

(13) $$l=\lambda_{2}E_{2}o$$

where $\lambda_{1}$ and $\lambda_{2}$ are the non-zero scale factors. Subtracting the equations in (12) and (13), we get

(14) $$(sE_{2}-E_{1})o=0,\;\mathrm{with}\;s={\lambda_{2}/\lambda }_{1}$$

For Equation (14), it is another equivalent form of Equation (11). We can use the MATLAB to solve it by the function polyeig(). There are three eigenvalues obtained since the matrix size is 3 × 3. According to the conclusion, two of them are identical and are different from the third one. The corresponding eigenvector of the third eigenvalue is the circle center [18].

## 3. Proposed Method

### 3.1. Phase-Shifting Pattern

Here we present the phase-shifting circular grating (PCG) patterns that encode the feature point into the intensity to calibrate the camera. While, for phase-shifting circular gratings, the images $I_{k}^{d}(x,y)$ are displayed on a monitor that can be expressed as [30]:

(15) $$I_{k}^{d}(x,y)=a+b\cos\left(\Phi^{d}(x,y)+\frac{2\pi (k-1)}{K}\right)$$

where *k* = 1, 2, …, *k*; Φ(x,y)=2πr(x,y)/T denotes the unwrapped phase; T denotes the period of the phase-shifting circular gratings; radius $r(x,y)=\sqrt{{\left(x-x_{0}\right)}^{2}+{\left(y-y_{0}\right)}^{2}}$ is the Euclidean distance between points (x,y) of the phase-shifting circular grating and its center (*x*_0_, *y*_0_); *a* and *b* can adjust the intensity of the patterns; Then, once they are captured by a camera and can be described as

(16) $$I_{k}^{c}(u,v)=A(u,v)+B(u,v)\cos\left(\phi^{c}(u,v)+\frac{2\pi (k-1)}{K}\right)$$

where A(u,v) is the average intensity, and B(u,v) is the intensity modulation of the phase-shifting patterns. When K≥3, A(u,v), B(u,v) and $\phi^{c}(u,v)$ can be obtained by the following,

(17) $$A(u,v)=\frac{1}{K}\sum_{k=1}^{K}I_{k}^{c}(u,v)$$

(18) $$B(u,v)=\frac{2}{K}\sqrt{{\left[\sum_{k=1}^{K}I_{k}^{c}(u,v)\sin\left(\frac{2\pi k}{K}\right)\right]}^{2}+{\left[\sum_{k=1}^{K}I_{k}^{c}(u,v)\cos\left(\frac{2\pi k}{K}\right)\right]}^{2}}$$

(19) $$\phi^{c}(u,v)=\arctan\frac{-\sum_{k=1}^{K}I_{k}^{c}(u,v)\sin2\pi k/K}{\sum_{k=1}^{K}I_{k}^{c}(u,v)\cos2\pi k/K}$$

With the phase-shifting patterns captured by the camera, the wrapped phase can be computed by Equation (19). 

**Figure 1:**
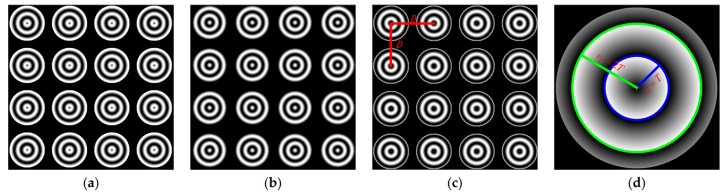
The phase-shifting circular patterns. (**a**–**c**) Images of the 4 × 4 PCG array patterns; (**d**) Wrapped phase of single PCG.

The pattern employed in this method consists of several identical circular gratings as shown in Figure 1a–c and we set *k* = 3 and *a* = *b* = 0.5. Since there is a linear relationship between the unwrapped phase and r(x,y), the points with same phase are distributed on a same circle. As zero-phase detection has the highest precision, we used a phase-shift technique to detect zero-phase points [31]. Especially, the zero-phase points are distributed on a circle with r(x,y) = *mT*, *m* = 1, 2, 3… In the literature, the points with Φ=2nπ, *n* = 1, 2, 3… are also called zero-phase points. Figure 1d shows that the zero-phase points are distributed on the blue and the green circle, of which the radius are *T* and 2*T* respectively. Meanwhile, they have one common circle center. *r_max_* which determines that the size of PCG is the maximum value of *r*(*x*, *y*). In order to ensure two complete PCG periods, a suitable *r_max_* should be chosen. 

PCG arrays are utilized to gain more circle centers as feature points for camera calibration due to one PCG only has one center. The array with *M* rows and *N* columns filled with uniform PCGs. The spaces between adjacent centers along the horizontal and vertical directions are equal, and their values are known. Let the space be *D_s_*. To avoid interference between adjacent PCGs, we let *D_s_* ≥ 2 *r_max_*. Through the above analysis, for a *M* × *N* PCG array, the zero-phase points distribute on *M* × *N* concentric circles and there are *M* × *N* feature points for calibration. According to perspective projection, the projection of the circle is ellipse [32]. Therefore, the imaged zero-phase points distribute on 2 × *M* × *N* ellipses. 

### 3.2. Feature Detection

As mentioned above, the imaged zero-phase points are distributed on 2 × *M* × *N* ellipses that are the projections of *M* × *N* concentric circles. Thus those ellipses curves would be computed accurately to locate the PCG centers which are used as the feature points. To start with, we provide a solution to separate the PCG from the array. A suitable threshold can be chosen to gain the binary mask Ω via the Equation (20). The Ω for the PCG array can be divided into *M* × *N* sub-masks for single PCG using the connected component labeling operation. The sub-image of each PCG therefore can be treated individually.

(20) $$\Omega =\left\{ \begin{array}{l} 1,\;if\;\left(I_{1}^{c}(u,v)+I_{2}^{c}(u,v)+I_{3}^{c}(u,v)\right)>threshold\\ 0,\;otherwise \end{array}\right.$$

According to Equation (19), the wrapped phase φ(u,v) of the proposed patterns can be computed as

(21) $$\varphi (u,v)=\arctan\left(\sqrt{3}\frac{I_{3}^{c}(u,v)-I_{2}^{c}(u,v)}{2I_{1}^{c}(u,v)-I_{2}^{c}(u,v)-I_{3}^{c}(u,v)}\right)$$

Then, the zero-phase points can be detected via the conventional Canny edge-detection algorithm because of 2π discontinuities. After that, the zero-phase points of each PCG are used to compute two ellipse curves by the least-squares ellipse-fitting algorithm [33]. A sub-mask can identify the wrapped phase of its corresponding PCG. The rough location of the imaged PCG center can be obtained as shown in Section 2.4. Since the edge detection operation could only extract the pixel ellipse, the zero-phase points should be refined to achieve sub-pixel accuracy. By using the constraint between the zero-phase and the radius [26], zero-phase point refinement easily achieves sub-pixel accuracy.

**Figure 2:**
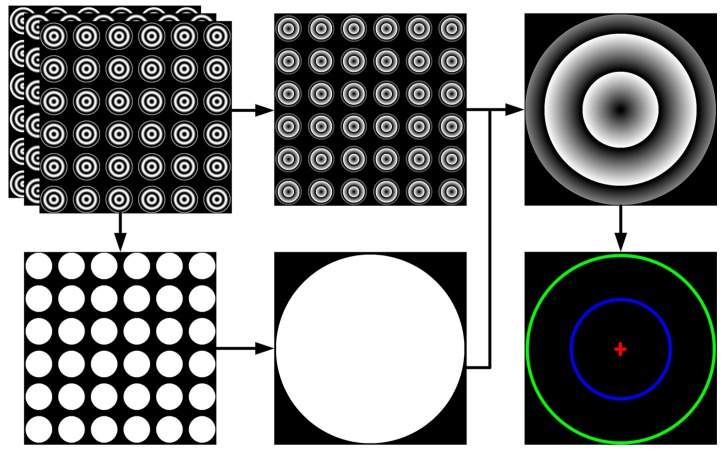
The map of the feature detection of the proposed method.

Once the zero-phase point sub-pixel optimization is solved, the high accuracy ellipse curves can be obtained by fitting ellipse with the least-squares method again. Repeating the step of circle center estimation, the real imaged center of PCG can be finally located. The whole map of feature detection of the proposed method is shown in Figure 2.

### 3.3. Sorting Feature Points

**Figure 3:**
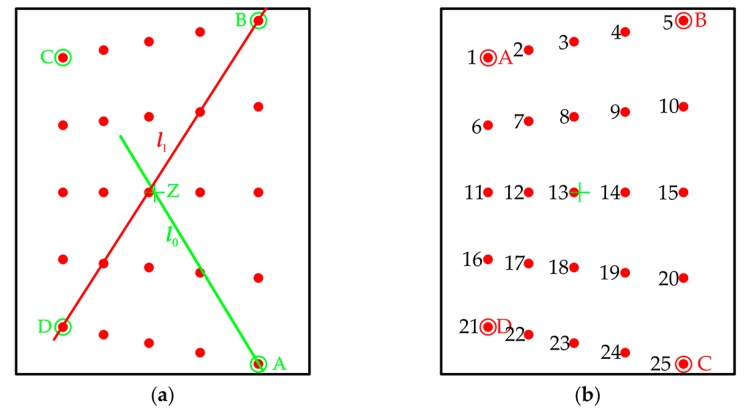
The schemes of the sorting algorithm. (**a**) To solve B and D using the straight line *l*_0_ and the final point C using the straight *l*_1_; (**b**) Reordering the vertexes and labeling the feature points.

Though the feature points are detected, the camera calibration should be conducted with them in a meaningful order. This section is therefore a crucial step for calibration, since it provides a solution to automatically label the feature points. Then a sorting algorithm is put forward to solve this problem and can be summarized as follows:

1. First of all, the centroid Z of those feature points is computed and the Euclidean distance between Z and the feature points can be used to identify one vertex. Meanwhile, the feature point whose distance is the longest can be regarded as the vertex, let it be A. Using point A and Z as the inputs, we can obtain a straight line $l_{0}:a_{0}u+b_{0}v+c_{0}=0\;(a_{0}>0)$.
2. We define $D_{0}=a_{0}u_{0}+b_{0}v_{0}+c_{0}$, and $\left(u_{0},v_{0}\right)$ is the coordinate of the feature point. The coordinate of each feature point is substituted into the equation to compute $D_{0}$. The $D_{0}$ is a signed float value, thus its maximum and minimum can be directly determined which point is B and D, respectively.
3. We can obtain another straight line $l_{1}:a_{1}u+b_{1}v+c_{1}=0\;(a_{1}>0)$ connecting point B and D. Repeating the step 2, $D_{1}=a_{1}u_{0}+b_{1}v_{0}+c_{1}$ can be calculated to locate the point C. Once the four vertexes are determined, we compute the sum of the row and column of each vertex. The minimum and maximum of the sum represent the upper-left point A and the down-right point C respectively. Then, the order of the vertexes can be refined.
4. Since the size of PCG array is known, the planar constraints can be used to order the feature points [34]. Finally, the calibration can be performed using the one-to-one mapping. The scheme of the sorting algorithm is presented in Figure 3.

## 4. Experiments and Results

In this section, we performed experiments with simulated and real images to verify the effectiveness and accuracy of the presented approach. All the experiments were conducted on a same computer, and the imaged centers of different targets were recovered in the same way as described in this paper.

### 4.1. Experiment on Simulated Images

In the computer simulation, the simulated images generated based on the principle of the ideal pinhole model are 1920 × 1280 resolution, where the distance between adjacent PCG centers *D_s_* = 375 pixels. However, the array size of virtual PCG array varied with the different simulations. The intrinsic parameters of the simulated camera are

(22) $$K=\left[\begin{array}{ccc} 2000 & 0 & 960\\ 0 & 2000 & 640\\ 0 & 0 & 1 \end{array}\right]$$

In the following simulations, we studied the impact of the PCG period and the number of PCGs on calibration accuracy and investigated the performance of PCG array against different noise levels. All the PCG array images used in the simulations are viewed by the simulated camera at 6 orientations. For each experiment, the process was repeated in 20 trials and the result was used to compute the error. The root-mean-square re-projection error (RMSE) was also computed to judge the influence of the above aspects to calibration.

**Influence of the number of PCGs.** In general, to increase the number of feature points is one of the way to improve the accuracy of calibration. This experiment is designed to study how the number of PCG of the proposed pattern impacts the calibration accuracy. Let the row and column of the PCG array be equal. The dimension of the PCG array is varied from 3 to 8 to change the number of feature points. The period of PCG is 45 pixels. For each number, the images were used to finish calibration with independent Gaussian noises with mean 0 and standard deviation 0.1 pixel. The errors were computed by comparing with the ground truth. 

**Figure 4:**
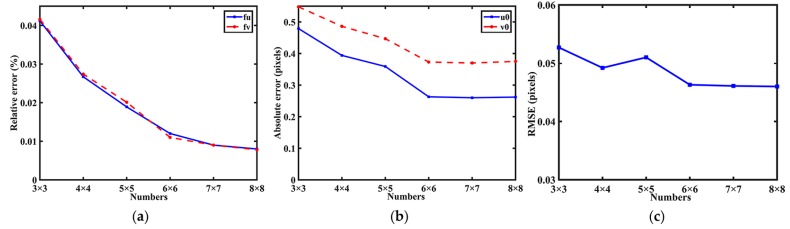
Errors regarding the number of PCGs. (**a**) Relative error for focal length; (**b**) Absolute error for principal point; (**c**) RMSEs with different number of PCGs.

As shown in Figure 4, the mean values of the errors and the RMSEs decrease as the number of PCGs increases. Thus sufficient feature points are essential in our method. Particularly, since the number of feature point was over 6 × 6, the RMSEs and the absolute errors in principal point are almost stable. 

**Influence of PCG period.** As mentioned above, the radius of zero-phase circles is relevant to the PCG period. To change the PCG period is to change its radius of zero-phase circles. To figure out the influence of PCG period to our method and select a suitable PCG period for the real scene, the experiment with different PCG periods was conducted. The virtual PCG arrays contained 5 × 5 uniform PCGs with *T* = 25, 30, 35, 40, 45, 50, 55, and 60 pixels. Meanwhile, Gaussian noise with zero-mean and standard deviation 0.1 pixel was added to the simulated images. 

**Figure 5:**
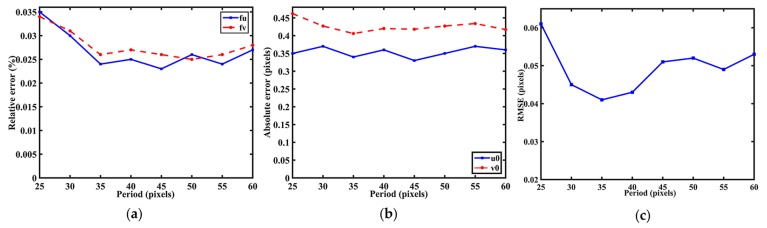
Errors regarding the period of PCG. (**a**) Relative error for focal length; (**b**) Absolute error for principal point; (**c**) RMSEs with different periods of PCG.

As it can be seen in Figure 5a,b, the accuracy changes slowly and the maximum differences of relative error in all the parameters are less than 0.05%. Theoretically, we can choose the period as large as we can to ensure the accuracy of center location. However, the size of monitor increases as the PCG period increases. The number of PCG must be reduced simultaneously, which could impact the final result. As shown in Figure 5c, though the RMSEs changed slightly with different period, there is still a suitable period to get higher accuracy. In the real condition, the PCG period is set at 35 pixels to ensure more PCGs displayed on the 1920 × 1080 Liquid Crystal Display (LCD).

**Figure 6:**
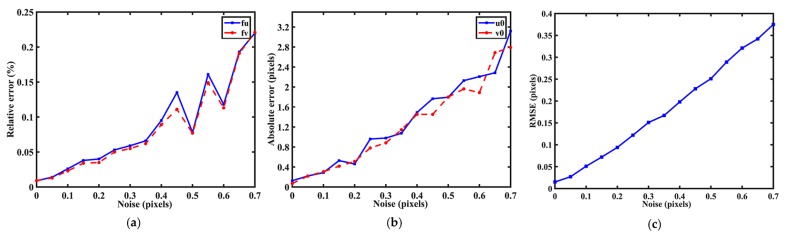
Errors regarding the noise level of the patterns. (**a**) Relative error for focal length; (**b**) Absolute error for principal point; (**c**) RMSEs with different noise levels.

**Influence of noises.** This experiment examined the influence of noises to the accuracy of location. The 5 × 5 PCG arrays with *T* = 45 pixels were employed to calibrate the simulated camera from six poses. The standard deviation of the Gaussian noises varied from 0 pixels to 0.7 pixels during the experiment. It can be seen from Figure 6a that the relative errors for focal length increase nonlinearly with the noises. When the noises are below 0.45 pixels, our method could show its robustness, but when the image noises are over 0.45 pixels, the errors decrease sometimes. Figure 6b,c shows that the absolute errors and RMSEs increased without regularity as the noise level increased and it cannot indicate the robustness of the proposed method to noise. So, it requires the higher precision of feature point extraction.

### 4.2. Experiment on Real Images

**Figure 7:**
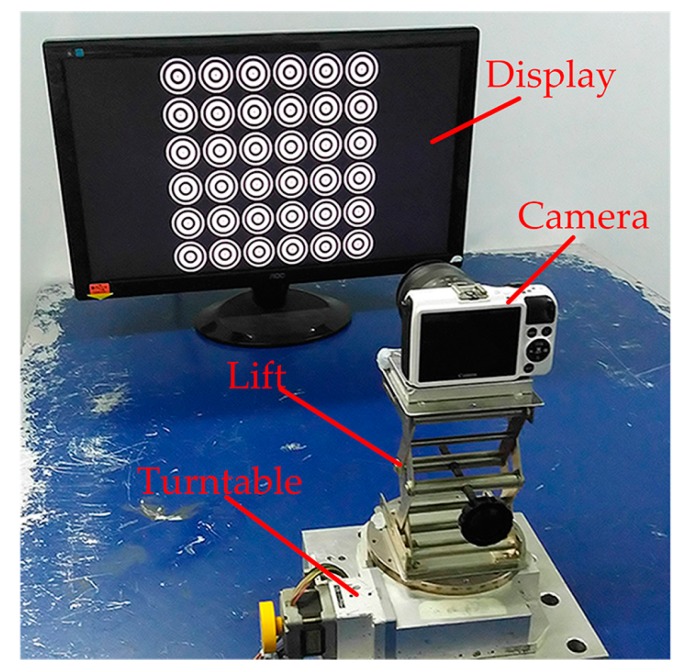
Set up of the real experiment.

To verify the performance of our method in real scene, a typical calibration system was set up as shown in Figure 7. The orientation and position of the camera can be adjusted by the device which consists of a turntable and a lift. The images were taken by a Canon EOS-M2 camera with a zoom lens, the resolution of the camera is 1920 × 1280. A LCD of Admiral Oversea Corporation with 1920 × 1080 resolution as the target to display the calibration patterns. To start with, the target was placed at a suitable distance from the camera, and the optical axis of the camera is perpendicular to the screen. The camera was fixed on the device, and a focal length was chosen to capture the sharp image of the pattern. Furthermore, the camera was controlled by a smartphone and the images can be obtained remotely.

Then, two experiments were designed and performed to verify the accuracy of the center location used in our method and the robustness to the defocus of the proposed method respectively. We used the same algorithm as in the simulations for camera calibration. All the operations were performed in the MATLAB.

#### 4.2.1. Accuracy Verification Experiment

The center of projection ellipse is directly regarded as the center of a spatial circle, which is improper under general perspective [15]. In the proposed method, the real imaged center of PCG was formulated as a concentric circle issue to solve, rather than directly using the center of projection ellipse. When the problem was treated as a concentric circle, the real projection can be computed using the method described in Section 2.4 and to verify the accuracy of the center estimation in our method, an experiment with our pattern was designed and performed. During the verification experiment, the rotation angle was changed by the turntable as shown in Figure 7, and the turntable was turned from 0 to 45°, and the images were captured every 15°. 

**Figure 8:**
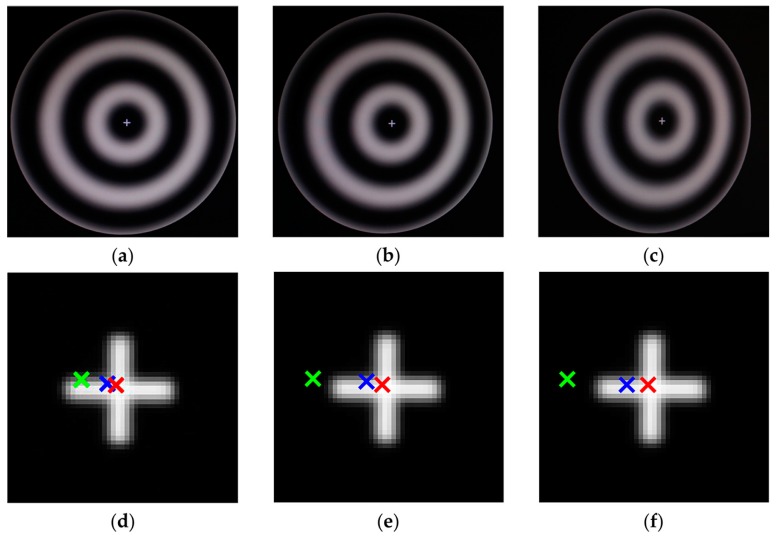
Captured images of different rotation angle and the images of enlarged region of PCG center. (**a**–**c**) The images of 15°, 30° and 45° respectively; (**d**–**f**) The enlarged region of PCG center of 15°, 30° and 45° respectively.

The experiment was carried out with one PCG patterns whose *T* = 200 pixels. Meanwhile, we made a spot on the PCG center (the white ‘+’ in Figure 8a–c). The captured images with 15°, 30° and 45° were shown in Figure 8a–c. For each position, the centers of inner (the blue ‘×’) and outer (the green ‘×’) ellipses were computed respectively as well as the imaged center located by our method (the red ‘×’). We enlarge the PCG center region in same magnification in Figure 8d–f, and the one-to-one match between the images and the center region is a–d, b–e and c–f. As it can be seen in Figure 8d,f, the centers of ellipses were farther from the real centers as the angle increased but the projection located by our method coincided well with the real projection. Then, we can conclude that the location accuracy of the method is more accurate than the method used in our previous work [25,26]. Thus, the result of this experiment verified the precision of our method and partly illustrated that our work is valuable and a contribution. The method is more suitable in the scene whose rotation angle is large. 

#### 4.2.2. Contrast Experiment with the Concentric Circle Array

In this experiment, we took the concentric circle array pattern and the PCG arrays for contrast experiments to illustrate the superiority of the later. Both of them contain 6 × 6 feature points, and their centers have same locations. The parameters of PCG arrays are: *a* = 0.5, *b* = 0.5, *T* = 35 pixels and *D_s_* = 180 pixels. Thus the radius of two concentric circles is 35 pixels and 70 pixels respectively. We properly adjust the focal length and the aperture of the camera to capture the images with three different defocus degrees. For each defocus degree group, we took images of the two targets from fifteen different orientations. The center of PCG and concentric circle is regarded as feature point. Meanwhile, the method to estimate the real imaged center of the two patterns is presented in Section 2.4. However, the sub-pixel ellipse extraction of a concentric circle is extracted by an optimized interpolation algorithm [20].

**Figure 9:**
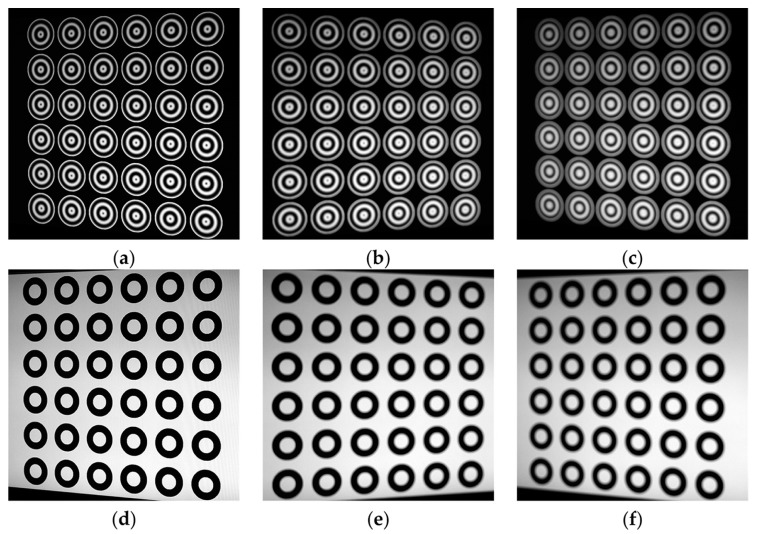
The captured images of different defocus degrees. (**a**–**c**) The images of PCG arrays of different defocus degrees respectively; (**d**–**f**) The images of concentric circle array of different defocus degrees respectively.

**Figure 10:**
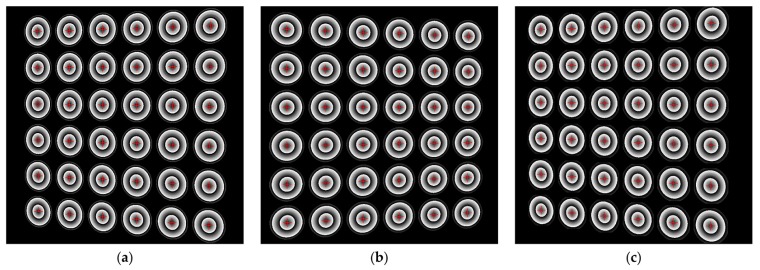
Wrapped phase with the calculated imaged centers of different defocus degrees. (**a**–**c**) The wrapped phase of Figure 9a–c respectively.

Figure 9 shows three sets of pattern images of different defocus degrees, and the first set images were captured almost in focus. Another two groups were slight and severe defocusing images respectively. As shown in Figure 10, the images of wrapped phase of PCG with detected feature points, and the red crosses denoted the imaged centers. We estimated the camera intrinsic parameters using the standard calibration method [34]. RMSE of feature points was used to evaluate calibration accuracy. The calibration results of three trials are listed in Table 1. As mentioned above, the first trial is to calibrate the camera using well-focused images. The RMSE in the presented method is a little smaller than concentric circle array. However, the difference between the two methods changed significantly as the defocus degree was severe. Calibration results show that the RMSE of the concentric circle array increased rapidly, but that of PCG was not. Such results indicate that our method can calibrate the camera with high accuracy even in the out-of-focus scene.

## 5. Conclusions

This paper has presented an accurate camera calibration method that is robust to defocus. By using the insensitivity to image defocusing of fringes, the proposed patterns for display are designed to be three phase-shifted circular grating arrays. The centers of PCGs are used as feature points. Since zero-phase points are distributed on concentric circles, the feature location problem is treated as a concentric circle issue. We estimate feature locations by the pole-polar relationship and algebraic operations rather than using the center of projection ellipse directly, such that the center can be located precisely. Our study gives a solution to the conventional troubles in using defocused images for camera calibration. We also present a sample sorting algorithm to label the feature points. Moreover, it requires just three frames at the same pose comparing with the other fringes patterns. The effectiveness of the proposed method has been validated by experiments with simulated and real images. The superiority allows the calibration using blurred images with a handheld camera. Thus, it is valuable for calibration of long-range vision systems.

## Figures and Tables

**Table 1:** Calibration result for real images using two patterns.

	Pattern	*f_u_*	*f_v_*	*u*_0_	*v*_0_	*k*_1_	*k*_2_	RMSE
**Trial 1**	PCG arrays	2748.012	2747.892	982.443	616.473	−0.010	0.099	**0.045**
	Concentric circle array	2745.681	2745.370	982.433	615.732	−0.012	0.103	0.054
**Trial 2**	PCG arrays	2732.675	2732.785	980.751	615.591	−0.012	0.105	**0.048**
	Concentric circle array	2721.353	2720.358	972.573	609.420	−0.041	0.182	0.136
**Trial 3**	PCG arrays	2674.015	2674.918	974.139	614.406	−0.047	0.174	**0.057**
	Concentric circle array	2680.222	2678.179	974.413	616.083	−0.060	0.243	0.179
